# Management of COPD and Comorbidities in COPD patients by Dispensing Pharmaceutical Care following Global Initiative for chronic Obstructive Lung Disease-Guidelines (GOLD guidelines 2020): A study protocol for a Prospective Randomized Clinical Trial^☆^

**DOI:** 10.1016/j.heliyon.2023.e21539

**Published:** 2023-10-25

**Authors:** Hafsa Kanwal, Shahzeb Khan, Gaber E. Eldesoky, Saima Mushtaq, Amjad Khan

**Affiliations:** a Department of Pharmacy, Quaid-i-Azam University, Islamabad, Pakistan; b Centre for Pharmaceutical Engineering, Faculty of Life Sciences, School of Pharmacy and Medical Sciences, University of Bradford, West Yorkshire, BD7, 1DP, UK; c Department of Chemistry, College of Science, King Saud University, Riyadh, Saudi Arabia; d Department of Healthcare Biotechnology, Atta-ur-Rahman School of Applied Biosciences (ASAB), National University of Sciences and Technology (NUST), Islamabad, Pakistan

**Keywords:** COPD, GOLD guidelines, Pharmaceutical care, COPD management, Role of pharmacist in disease management

## Abstract

COPD (chronic obstructive pulmonary disease) is a medical condition that encompasses several chronic, progressive, and severe respiratory illnesses, such as emphysema and chronic bronchitis. COPD is the 4th most deadly disease in the world and its prevalence is expected to increase. Despite the abundance of information on the disease's etiology, pathophysiology, and treatment possibilities, it has long been underdiagnosed and underreported for a long time, particularly in developing countries. The symptoms of COPD result in significant impairments and significant impact on quality of life. COPD is the third leading cause of death in Pakistan. According to the published literature, COPD has been found to be associated with a serious economic burden, either the direct cost to healthcare systems in the form of frequent hospital admissions or indirect costs to patients suffering from COPD. Despite the availability of excellent medication, COPD treatment goals are frequently not achieved resulting in poor management of COPD. The recent studies revealed that due to the missing role of Pharmacists in most of the public sector hospitals of Pakistan, the COPD disease management protocols are not being properly followed. Pharmacists can help the healthcare system by implementing these management protocols that focus on patient education about the disease, prescribed medications, and proper inhalation techniques. Furthermore, the pharmacists as an effective healthcare's team member properly educate the patients about the ongoing assessments and their willingness to follow treatment recommendations and quit smoking, while referring them to smoking cessation programs as needed, following the GOLD guidelines. This aim of this clinical trial is to evaluate the impact of implementing standard treatment guidelines and the role of pharmacists in implementing GOLD guidelines for COPD management.

## 1 Introduction

The primary manifestation of COPD is restricted airflow in the airways and is most commonly associated with an atypical allergic reaction in the lungs triggered by harmful environmental substances. This condition also leads to decreased lung flexibility or emphysema [1,2]. The current state of is alarming as it affects 2.7 million individuals annually. COPD is predicted to become the third most common disease by 2030, if left untreated or without effective preventive measures [1,3,4]. The literature suggests that COPD's annual mortality rate from COPD could exceed the combined death rates of lung cancer and breast cancer [5,6]. Smoking is the primary underlying factor in COPD, accounting for around 85–90 % of all COPD cases [7]. The quality of life negatively impacted by COPD, which increases healthcare resource utilization and spending. While a lack of proper pharmacotherapy may impair patient and healthcare outcomes, overtreatment should also be avoided. Especially indications for inhaled corticosteroids (ICS) have become fewer, due to their unfavorable cost-benefit-ratio, with regard to serious adverse events and the superior effectiveness of long-acting bronchodilators (BD) in decreasing exacerbations [8]. COPD is becoming a significant global health challenge, with a particular emphasis in developing regions. In addition to the smoking as a risk factor, the lack of disease awareness among the general population and the inadequate implementation of treatment guidelines also play a role in contributing to the prevalence of COPD [9]. To ensure that COPD patients receive adequate care, it is crucial to adhere to standardized treatment protocols. The Global Initiative for Chronic Obstructive Lung Disease (GOLD), established in 2001, plays a pivotal role in enhancing the diagnosis, management, and prevention of COPD, to improve the treatment outcomes for COPD patients. Each year, a new report is generated through an extensive examination of published studies, to enhance the practices of healthcare providers in COPD management. These GOLD reports are widely recognized as crucial, evidence-based reference materials for implementing effective management protocols and represent the current guidelines for the care of individuals with COPD [10]. The GOLD guidelines, which were initially introduced in 2011, were created to provide healthcare providers with the most suitable recommendations for diagnosing and managing COPD patients. This handbook has been revised and updated with the new guidelines in the years including 2006, 2011, 2017 and 2021. The updated handbook with the new indispensable instruments has received noticeable attraction by the clinical practicians across the world [11,12]. Taking into account the significance of COPD guidelines for disease management in many different countries, clinicians treating various patient populations exhibited poor adherence to GOLD guidelines recommendations [[13], [14], [15], [16], [17], [18], [19], [20], [21], [22]], specifically over-treating the patients [20]. The World Health Organization (WHO) states that the factors affecting the adherence of pulmonology specialists to guidelines are highly intricate, encompassing not just the physician but also factors related to the patient, the disease itself, and social aspects [21,23]. Regardless of the obstacles, patients' clinical outcomes are negatively impacted by inadequate adherence which increases the risk of unforeseen acute exacerbations of COPD [24,25]. Enhanced adherence can lead to clinical and functional advantages, as well as economic benefits that reduce both direct and indirect healthcare costs [16,[26], [27], [28], [29]].

As, COPD is the 3rd most prevalent non-communicable disease, there is limited data available on therapeutic and economic outcomes related to COPD in Pakistan [5]. In Pakistan, there is a lack of data on the clinical and economic outcomes of Medicare beneficiaries with COPD who reside in long-term care (LTC) facilities [30]. Despite the benefits of guideline-complying therapy, various cross-sectional studies demonstrated that GOLD-implementation was suboptimal, likely due to physicians’ poor familiarity with guidelines. It is evident that there is a lack of knowledge about clinical realization of recommended therapy settings [31,32].

Pharmacists have the ability to provide education on disease, medication treatment, and healthcare guidelines and policies to either healthcare practitioners or patients. Therefore, they can collaborate with physicians to implement the COPD GOLD guidelines. The aim of this study is to implement the recommendations of the GOLD guidelines in healthcare settings in Pakistan.

### 1.1 Objectives

A. **Research Hypothesis:** Integrating the standardized protocols outlined in the GOLD guidelines into clinical practices will enhance COPD management in Pakistan.
B. **Study objectives:** The objective of this research is to evaluate the enhancement of COPD management by implementing standardized treatment guidelines, to improve treatment outcomes, slow down disease progression and enhancing the quality of life. This endeavor is expected to have a positive impact by reducing the frequency of acute exacerbations, emergency visits, and hospital stays. Therefore, it will indirectly result in alleviating the healthcare burden of COPD on the healthcare system in Pakistan.

### 1.2 Trial design

In order to effectively carry out this ongoing clinical trial, an exploratory randomized controlled study design with three arms is necessary. These arms include Group A (receiving usual therapy), Group B (receiving Pharmaceutical Care, encompassing pharmacological and non-pharmacological treatments in accordance with GOLD guidelines) and Group C (receiving Usual Treatment along with an educational intervention). Patients will be randomly assigned to one of these three arms based on their medical record numbers, with 1:1:1 allocation ratio. The trial was meticulously designed to comply with the Standard Protocol Items: Recommendations for Interventional Trials (SPIRIT), and the checklist of SPIRIT guidelines is included in (Appendix A).

## 2 Methods: participants, interventions, outcomes

### 2.1 Study setting

A. Selection of the country: Pakistan, being a developing nation with limited resources, faces challenges in adequately addressing a wide range of health issues. In many cases, the management of chronic diseases does not adhere to established treatment protocols, leading to an escalation in healthcare costs.
B. **Selection of the disease:** COPD, which ranks as the 3rd leading cause of death in developing countries, including Pakistan, has been reported a major healthcare debacle with high negligence. Hence, we have selected COPD as the focus of our study to assess the response to intervention-based disease management following the standard treatment guidelines.
C. **Study design:** Patients recruitment for the trial will be based on strict compliance with both inclusion and exclusion criteria**.** Once they have signed the participant's consent form, they will be randomly assigned to one of three groups: the first group will be the treatment group 1, the second group will be the treatment groups 2, and the third group will serve as the control group. The first treatment group will receive an educational intervention, which will consist of pharmacist counseling that covers information about the disease, medications, and lifestyle modifications. On the other hand, the second treatment group will receive pharmaceutical care, which includes pharmacological interventions and educational interventions in accordance with the GOLD guidelines. The usual care will be provided to the participants in the control groups. The effectiveness of these interventions will be assessed through several measures which include: a) The number of acute exacerbations and hospital emergency visits will be documented based on patients' medical records, including prescriptions and lab reports, throughout the study b) Disease progression will be evaluated using spirometry c) The quality of life of the patients will be assessed using the St. George's Respiratory Questionnaire [33].
D. **Description of study settings:** The study will take place within the Medical Outpatient Department (OPD) of Holy Family Hospital, Rawalpindi Medical University in Pakistan, to recruit a total of 120 participants diagnosed with COPD. Following the completion of the clinical trial, the data will be accessible on the trial registry where it was initially registered, as well as on other scientific literature publishing platforms.
E. **Informed consent:** Patients will be provided with the informed consent before any study procedures occur.

### 2.2 Eligibility criteria

Patients who meet all the specified inclusion criteria and none of the stated exclusion criteria will be eligible for participation. The enrollment period is scheduled to last 24 weeks for assessing primary outcomes and 48 weeks to assess secondary outcomes.

A. **Diagnostic criteria:** The study's focus will be on individuals who experience dyspnea and chronic cough with sputum production as their target population. Clinical assessment, which includes spirometry will however be used to establish the diagnosis. To confirm the persistent airflow limitation, the Forced Expiratory Volume (FEV1)/Forced Vital Capacity (FVC) ratio must be less than 0.70.
B. **Inclusion Criteria:** Patients who are eligible to participate in the trial must meet all of the following criteria at the time of randomization: **a)** Patients should exclusively receive care in the Outpatient COPD wards **b)** They must have a confirmed diagnosis of COPD by the hospital consultant, which includes a FEV1/FVC ratio of less than 0.70 compared to the predicted normal value, and a history of two or more acute exacerbations (emergency visits) within the past 6 months to 1 year **c)** A comprehensive diagnosis will be conducted to assess any co-morbidities **d)** Patients deemed suitable for trial entry by the consultant and who possess a clear understanding of the study's significance, demonstrate good compliance with the researcher's observations and evaluations, willingly accept participation in the clinical trial, and complete the clinical consent form, will be included in this study.
C. **Exclusion Criteria:** Patients who have mobility issues, confusion, disorientation, terminal illness or congestive heart failure will not be included in the study. Additionally patients who are currently enrolled in any rehabilitation programs or seeking consultation with a clinical pharmacist or pulmonary physiotherapist will be excluded [34,35].

### 2.3 Interventions

#### 2.3.1 Interventions for each group with sufficient details

The clinical trial will be divided into three parts as follows:

**Arm 0**: Patients in this group won't receive any special treatment and will continue to receive their usual care.

**Arm 1:** The principal investigator will exclusively provide educational intervention to COPD patients in this group. The intervention will involve disease education, proper medication usage, and lifestyle modifications, which will include dietary and exercise recommendations. The medications prescribed by their physician will continue to be given to the patients in this group.

**Arm 2:** The standard COPD GOLD guidelines will be followed to administer pharmaceutical care as follows:

Step 1 The recommended standard diagnostic procedures outlined in the guidelines will be used for patients to undergo diagnosis. The guidelines will categorize them into the appropriate GOLD stages based on their diagnosis. In addition, there will be monitoring to detect any potential co-morbidities.

Step 2 Pharmacological treatment will be given based on the diagnosed stage of the disease, as well as non-pharmacological interventions like flu vaccines.

Step 3 Educational intervention in the form of a 30-min face-to-face patient counseling session will also be provided, following the guidelines as mentioned in the first arm.

#### 2.3.2 Study activities

1. Patients will be registered at the outpatient department of Holy Family Hospital in Rawalpindi, Pakistan, with the principal investigator and a physician administering the interventions.
2. Interventions will be administered to both groups when patients are enrolled.
3. Patients will be asked to assess their adherence during follow-up visits every two months. Patients interviews and a review of their prescriptions and medications will be used to evaluate treatment compliance.
4. Interventions will be given only once, however, the final evaluation which include, the disease state, quality of life, and the number of acute exacerbations during the study period, will be conducted after six months.

#### 2.3.3 Criteria for discontinuing or modifying allocated interventions for a given trial participant

The treatment regimen will be modified if the patients' condition worsens, and the number of emergency visits exceed the specified threshold.

#### 2.3.4 Strategies to improve adherence to interventions

After enrollment, there will be in-person reminder session that will be conducted every two months during each follow-up appointment. The following components will be covered during these sessions.

a) Emphasizing the significance of adhering to the study guidelines regarding medication adherence, providing instructions on medication administration, including dosage regimen and storage, impact of medication adherence and guidance on dose adjustment if a dose is missed.
b) Subsequent sessions will be held during follow-up visits. Participants will be evaluated objectively and subjectively to assess their disease status and quality of life during these sessions. A brief discussion will be held on the reasons for missed doses and simple strategies to enhance adherence. The discussion will focus on linking intervention adherence to daily routines and considering the impact on health-related quality of life and the frequency of emergency visits.

#### 2.3.5 Outcomes

Primary outcome [1]:

Quality of life assessed using the St. George's Respiratory Questionnaire.

Timepoint [1]:

After 24 weeks/6 months of commencement of intervention the outcomes will be assessed.

Primary outcome [2]:

No. of acute exacerbation or No. of emergency hospital visits will be counted from patients’ medical record (prescriptions, lab reports etc.)

Timepoint [2]:

24 weeks/6 months after interventions.

Primary outcome [3]:

Spirometry will be used to assess disease progression. Forced Expiratory Volume (FEV1) will be measured for 1 s measured before and after intervention to study the impact of interventions.

Timepoint [3]:

The disease will be assessed after 24 weeks or 6 months of intervention.

Secondary outcome [1]:

The comparison will involve comparing the number of acute exacerbations (no. of emergency visits) and mortality rate of all study groups.

Time point [1]:

After 1 year of interventions.

### 2.4 Participant timeline

**Fig. 1:**
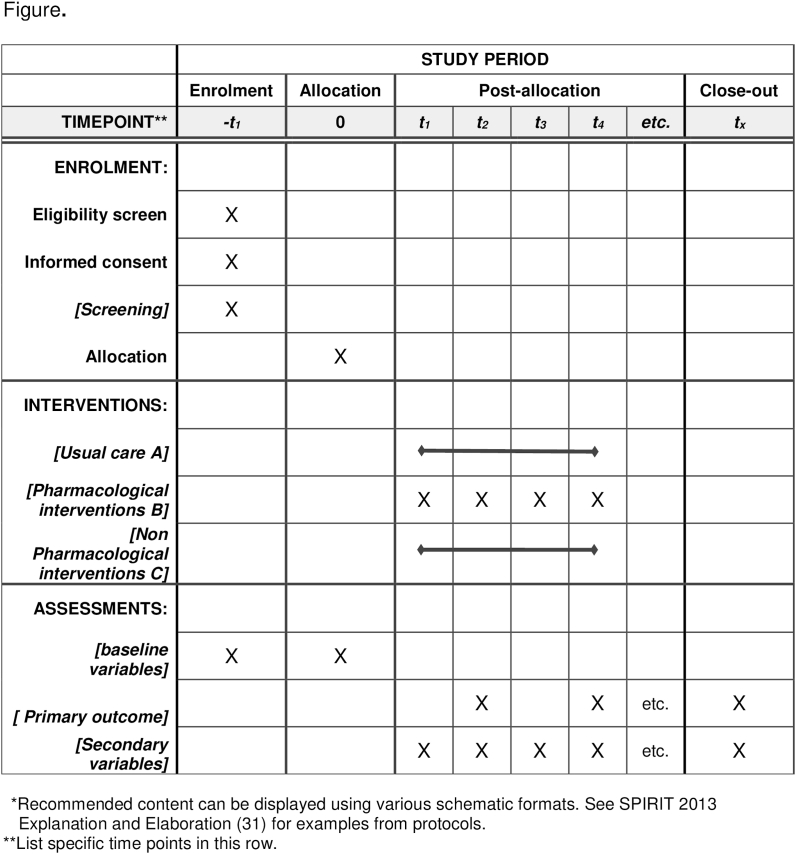
Patient timeline according to the SPIRIT guideline following SPIRIT guideline 2013.

See Fig. 1: Patient timeline according to the SPIRIT guideline following SPIRIT guideline 2013.

a. Measures to enhance the patients' adherence to the clinical trial:

Each patient's drug quantity and date of drug ingestion will be documented. During each visit, the patients will be expected to talk about the missing medications, which will be documented by the principal investigator.

b. Assessment of Clinical trial progress time:

The participants will be scheduled for an assessment of their adherence to the interventions, particularly at the two-month follow-up intervals. Data will be collected about patient quality of life, the number of acute exacerbations, and disease progression during these assessments. Spirometry tests will be conducted during each visit to monitor these parameters.

### 2.5 Sample size estimation and recruitment target

The primary goal of this feasibility study is to gather eligibility and recruitment data to provide a precise estimate of the required sample size for a future trial. To achieve the specified participant count, a realistic recruitment strategy will be utilized, based on findings from previously published clinical trial studies. We anticipate enrolling 120 patients for this study, and we have a contingency plan that will cover a dropout rate of 5 %–10 %.

### 2.6 Recruitment

**Fig. 2:**
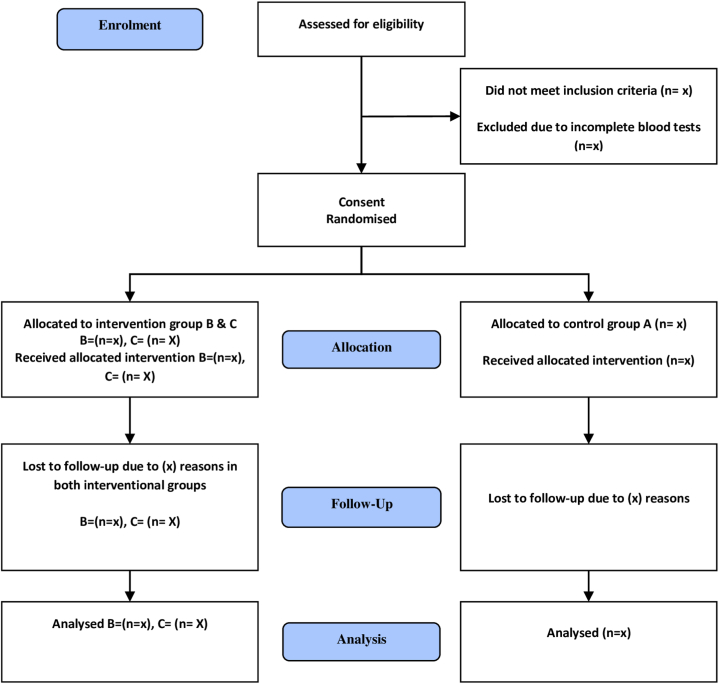
CONSORT Flow Diagram of screening, randomization and follow-up.

The principal investigator will undertake the recruitment of participants from Holy Family Hospital in Rawalpindi, Pakistan, following the screening, randomization, and follow-up process outlined in Fig. 2 of the CONSORT Flow Diagram. Participants in the trial will be given a brief overview of the trial's objectives, methodologies, potential side effects, and advantages. The following two primary strategies will be implemented to ensure an adequate number of participants during the enrollment phase, which will last for 24 weeks.

1. Disseminating recruitment materials in prominent hospital locations, including the outpatient lobby and ward entrances.
2. In cases where potential participants are identified within the hospital wards, either the hospital's inpatient staff or the principal investigator will approach them to gauge their interest in participating

### 2.7 Methods: assignment of interventions (for controlled trials

A. **Sequence Generation:** Participants will be assigned randomly to either the control or experimental groups, following a 1:1:1 allocation ratio determined by a computer-generated randomization schedule.
B. **Confidentiality:** The personal medical records of the subjects will only be granted access to by the principal investigator of the clinical trial.

The principal investigator will also be required to sign an “Investigator Statement” or “Confidentiality Agreement” affirming their commitment to maintaining confidentiality. Data handling will strictly adhere to a “data anonymization” approach, ensuring that any information that could reveal the specific identity of the subjects is excluded. The allocation sequence will be generated by the principal investigator and the hospital's health care team, participants will be enrolled, and they will be assigned to interventions.

### 2.8 Methods: data collection, management, analysis

#### 2.8.1 Data collection methods

A. **Data collection methods:** Prior to enrolling patients into either the control or experimental groups, a baseline assessment will be conducted to evaluate the patients' disease status, including co-morbidities, quality of life and their knowledge about the disease. Pre-validated questionnaires will be utilized in this assessment. To compare the interventional groups and control group, the same parameters mentioned above will be assessed at each follow up.
B. **Retention**: To ensure their continued participation in this study, patients will be given medications as well as other health care services.

##### 2.8.1.1 Data management

The principal investigator will use pre-validated questionnaires to collect all the data in collaboration with the patients' pathology records. This data will be electronically recorded for future analysis and interpretation. Data collection will happen both during the baseline and after each follow-up assessment. The participant files will be systematically arranged in numerical order and stored securely in an easily accessible location. Following the conclusion of the study, the participant files will be kept in storage for a duration of 3 years.

#### 2.8.2 Statistical methods – outcomes

The intervention groups will be compared to the control group in our primary analyses. The chi-squared test will be used to evaluate binary outcomes, while continuous outcomes will be evaluated using the T-test. In order to analyze subgroup, regression techniques will be employed and appropriate interaction terms (i.e., subgroup × treatment group) will be incorporated. The use of logistic regression for binary outcomes and linear regression for continuous outcomes will result in multivariable analyses. The residuals will be analyzed to assess model assumptions and goodness-of-fit.

We will utilize Kaplan-Meier survival analysis for time-based endpoints like mortality, followed by multivariable Cox proportional hazards modeling to adjust for baseline variables. During the statistical analysis phase, SPSS software version 22 will be used to code and enter the for preliminary and 6-month assessments.

## 3 Discussion

The most vital role in the healthcare team is of the pharmacist, who can play his role in the management of COPD by implementing standard guidelines focusing on patient counseling about the problem and health care utilization along with the correct medication regimen [36]. To assess the impact of pharmaceutical care on COPD patients, randomized controlled clinical trials are necessary, following the guidelines named GOLD guidelines 2020. This study will provide an insight to check the improvement in patients’ quality of life and decrease the economic burden of the healthcare department, working in collaboration with other healthcare team members in Pakistan. The aim of this study is to establish a relationship between the physician and pharmacist, enabling them to provide high quality care while making the decisions for the patient. This would be accomplished by providing pharmaceutical care in one of the interventional groups of the study. Additionally, the impact of educational intervention on establishing the effective disease course is imperative to be assessed. This will solely highlight the role of pharmacist and how his/her role will improve the disease state. It is not an easy task to develop a treatment protocol and practice in healthcare facilities, particularly in countries in like Pakistan. The success of this clinical trial will provide an insight into the impact of pharmaceutical care as well as educational interventions on improving COPD management.

## Research ethics approval

This study is reviewed and approved by IRBs/ECs present in Appendix B under the No. #BEC-FBS-QAU2021-269 is with respect to scientific content and compliance with applicable research and human subject regulations.

## Informed consent

The study participants will be thoroughly informed by the consent form about all aspects of the trial by the principal investigator. Before any study-specific treatments or examinations are performed, each patient will give written informed permission after learning about the study's goals, methodology, expected benefits, and potential risks. The patient's readiness to take part in this trial will be updated in the clinical trial registry under the number ACTRN12622000234718 after taking the permission a specific consent form that the patient will be signed. The original consent form will be kept by the investigator, and a copy will be given to the patient. Patients will also be informed that they have the option to refuse participation into the study and to withdraw at any moment without affecting their future treatment. Patients will be given written and/or oral information regarding the research in simple words; nevertheless, only patients who are able and willing to read and interpret written and verbal instructions will be enrolled in the trial, as described in the study inclusion criteria.

## Consent or assent

Who will obtain informed consent or assent from potential trial participants.

A signed consent will be obtained from every participant in the ancillary study by the principal investigator.

## Confidentiality

The purpose of getting personal information is to adhere the patient in the clinical trial and once the study will be completed each patient will be given a specific code hiding his/her original identification.

## Access to data

Only the principal investigator and primary sponsor will have the access to the data until it will be published.

## Data availability statement

Data included in article/supp. material/referenced in article.

## CRediT authorship contribution statement

**Hafsa Kanwal:** Conceptualization, Investigation, Methodology, Writing – original draft. **Shahzeb Khan:** Writing – review & editing. **Gaber E. Eldesoky:** Methodology, Resources, Software. **Saima Mushtaq:** Writing – review & editing. **Amjad Khan:** Conceptualization, Data curation, Formal analysis, Methodology, Project administration, Resources, Supervision, Writing – review & editing.

## Declaration of competing interest

The authors declare that they have no known competing financial interests or personal relationships that could have appeared to influence the work reported in this paper.
