# Supplementary material for: Management of COPD and Comorbidities in COPD patients by Dispensing Pharmaceutical Care following Global Initiative for chronic Obstructive Lung Disease-Guidelines (GOLD guidelines 2020): A study protocol for a Prospective Randomized Clinical Trial
Source: Heliyon. 2023 Oct 25;9(11):e21539. doi: 10.1016/j.heliyon.2023.e21539 (PMC10628705; doi:10.1016/j.heliyon.2023.e21539)
Supplement: Multimedia component 3 [file mmc3.pdf]

HIGHER EDUCATION COMMISSION

Research and Development Division

No: Ref No. 20-14413/NRPU/R&D/HEC/2021 2021 Creation Date: 01.04.22

1. Dr. Amjad Khan,
   Assistant Professor,
   Pharmacy,
   Quaid-i-Azam University, Islamabad

SUBJECT: PROVISIONAL AWARD UNDER National Research Program for Universities- NRPU

Reference research project No 14413 entitled "Evaluation and Management of COPD and other Comorbidities in COPD Patients by Dispensing Pharmaceutical Care following the GOLD guidelines 2020: A Prospective Clinical Trial" having total cost of Rs. 1373100/- and project duration of 24 months, submitted for funding by Dr. Amjad Khan, Quaid-i-Azam University, Islamabad, (the Principal Investigator).

The Higher Education Commission (HEC) hereby awards Rs. 1373100/- (one million three hundred seventy three thousand one hundred) to the Quaid-i-Azam University, Islamabad (the Grantee) for the above mentioned project subject to the provision of following documents:

a. Final revised proposal after incorporating reviewer's comments in letter & sprit, duly signed/endorsed by HoD, Director ORIC, Head of Institution (Vice Chancellor/Rector)

b. A certificate duly signed by HoD and PI stating that reviewer's comments have been incorporated in the revised proposal (specimen attached)

c. Revised Implementation Plan of the project after incorporating reviewer's suggations if any.Gantt Chart of Procurement Plan of the project

You are, therefore. requested to execute an agreement on stamp paper as per attached specimen and the same may be furnished to this office within 15 days of the issuance of this award. The award shall stand cancelled upon failure on your part to submit the agreement with in the stipulated period of time. This award is subject to the Terms and Conditions of the Grant outlined in the specimen Grant Agreement.

Detailed breakup of budget is as under:

Budget Element	Total Budget PKR
PI: Dr. Amjad Khan Assistant Professor Pharmacy Quaid-i-Azam University, Islamabad:	300000
Co-PI: Prof. Dr. Gul Majid Khan Professor	200000
PhD Student	144000
Total Equipment Cost	250000
Total Consumable Supplies	200000
Total Services Cost	50000
Total Travel Cost	50000
Total Budget (Direct Cost) - A	1194000
Total Overhead Cost	179100
G. Total Budget	1373100

Disbursement plan for the release of funds are as under

Item	%	Amount in Rs.	Remarks
1st Installment	60%	823860	Upfront payment
2nd Installment	20%	274620	will be released subject to the satisfactory review of 1st Annual Report
Final Installment	20%	274620	Will be released after successful completion of the project
Total Approved Cost		1373100	

Sincerely

RGM Section,
R&D Division,
Higher Education Commission,
Islamabad

Copy for Information to:

I. Vice Chancellor, Quaid-i-Azam University, Islamabad
II. The Director Finance, Quaid-i-Azam University, Islamabad
III. The Director ORIC, Quaid-i-Azam University, Islamabad
