# Supplementary material for: Management of COPD and Comorbidities in COPD patients by Dispensing Pharmaceutical Care following Global Initiative for chronic Obstructive Lung Disease-Guidelines (GOLD guidelines 2020): A study protocol for a Prospective Randomized Clinical Trial
Source: Heliyon. 2023 Oct 25;9(11):e21539. doi: 10.1016/j.heliyon.2023.e21539 (PMC10628705; doi:10.1016/j.heliyon.2023.e21539)
Supplement: Multimedia component 4 [file mmc4.pdf]

A Disease-specific Questionnaire (A)

Number:	Sex:☐ Female ☐Male	Date of birth:
Marital status：☐ Unmarried ☐ Married ☐ Divorce ☐ Widowhood		
Home address:	Telephone number(at least two):	
Educational level：①Primary school or below②Middle school③High school④College or above		
Smoking history: pack a day, years	Quit smoking or not: ☐ Yes, years ☐ No	
**Occupational exposure history**	**Exposed or not:**☐ Yes ☐No	
①—Silicious dust	②—Coal dust	③—Graphite dust
④—Conductex	⑤—Asbestos	⑥—Talc dust
⑦—Cement dust	⑧—Mica dust	⑨—Clay dust
⑩—Aluminum dust	⑪—Welding dust	⑫—Foundry dust
⑬—Cotton dust	⑭—Metals and their complexes	⑮—Irritant chemicals：oil paint
⑯—Hard metal (wolfram/ titanium)	⑰—others	
Exposure duration：Years, hours/per day		
**Biofuel exposure historyExposed or not:** ☐ Yes ☐No		
Types: ☐ faggot☐ wood☐ charcoal☐ crops rod☐ animal waste☐ cooking ☐ others		
Exposure duration：Years, hours/per day		

**Lung functionHeight:cm** **Weight:kg**				Blood eosinophils
FEV$_1$/pre%	pre：	FEV$_1$/FVC%	pre：	Numbers of EO：<br>10^9/L
	post：		post：	
FEV$_1$(ml)：		Improvement rate in FEV$_1$：		EO：%

**X-ray☐ Yes☐ Nonumbers of X-ray:Date:**
Results：
**CT ☐ Yes☐ Nonumbers of CTDate:**
Results：

**FeNO☐ Yes**	**☐ No**

One pack cigarette including 20 cigarettes

ADisease-specific Questionnaire (B)

**CAT（Scored on 0 to 5 scale where 5 is the worst）**		
I never cough	⓪①②③④⑤	I cough all the time
I have no phlegm (mucus) on my chest at all	⓪①②③④⑤	My chest is full of phlegm (mucus)
My chest does not feel tight at all	⓪①②③④⑤	My chest feels very tight
When I walk up a hill or a flight of stairs I am not out of breath	⓪①②③④⑤	When I walk up a hill or a flight of stairs I am completely out of breath
I am not limited to doing any activities at home	⓪①②③④⑤	I am completely limited to doing all activities at home
I am confident leaving my home despite my lung condition	⓪①②③④⑤	I am not confident leaving my home at all because of my lung condition
I sleep soundly	⓪①②③④⑤	I do not sleep soundly because of my lung condition
I have lots of energy	⓪①②③④⑤	I have no energy at all

**mMRC**	
**0**	No dyspnea except on strenuous exercise
**1**	Short of breath when walking up a short hill
**2**	Dyspnea limits walking pace (slower than others of same age) and stops to catch breath
**3**	Stops to catch breath after walking 100 yards (or meters) or after a few minutes on level ground
**4**	Dyspnea prevents leaving house and performing activities of daily living

Number of exacerbations in the past year:, ① numbers of moderate exacerbations (treated with SABDs plus antibiotics and/or oral corticosteroids): _______,duration:②numbers of severe exacerbations (patient requires hospitalization or visits the emergency room): , duration:. (a COPD exacerbation was calculated at 14-day intervals)

Diagnosis：☐Asthma☐Emphysema☐COPD ☐ACOS or ACO

☐AECOPD ☐Bronchiectasia☐Tuberculosis（TB）☐Lung cancer☐Others

Comorbidities:

1)High blood pressure	2) diabetes
3) dyslipidemia	4) heart failure
5) coronary artery disease	6) atrial fibrillation
7)acute and chronic renal failure	8) cancer
9) osteoporosis	10) stroke
11) Degenerative disease of the central nervous system	Any other

**Therapies**	**Treated with drugs or not: ☐ Yes☐ No**						
	**Trade name**	**Ingredient**	**Specifications**	**Dose**	**Frequency**	**Trade name**	**Ingredient**
☐	Ventolin®	Salbutamol Sulphate Aerosol	100ug			☐Anoro®	Umeclidinium bromide and vilanteroltrifenatate
☐	Onbrez®	Indacaterol maleate	150ug			☐Ultibro®	Indacaterol Maleate and Glycopyrronium
☐	Atrovent®	Ipratropium Bromide Aerosol	20ug			☐RELVAR®	Fluticasone Furoate and VilanterolTrifenatate
☐	Tianqingsule®	Tiotropium Bromide	18ug				
☐	Spiriva®	Tiotropium Bromide	18ug				
☐	Spiriva®Respimat	Tiotropium Bromide	2.5ug				
☐	Seretide®	Salmeterol/fluticasone	50/100				
☐	Seretide®	Salmeterol/fluticasone	50/250				
☐	Seretide®	Salmeterol/fluticasone	50/500				
☐	Symbicort®	Budesonide/formoterol	160/4.5				
☐	Symbicort®	Budesonide/formoterol	320/9				
☐	Foster®	Beclomethasone/formoterol	100ug				
☐	Flixotide®	Fluticasone propionate	125ug				
☐	Xianding®	Ciclesonide	100ug				
☐	Muerchang®	Budesonide Inhalation Powder	200ug				
☐	Theophyllines		☐Aminophylline		☐ Theophylline		
☐	Phosphodiesterase		☐ Roflumilast				
☐	Others: Montelukast						
☐	Other therapies	☐Oxygen therapies at home☐ Noninvasive ventilationat home☐Pulmonary rehabilitation ☐ ☐Vaccination☐ Lung transplantation☐ Others					

<u>St. George Quality of life Questionnaire</u>

Part 1

Question 1: I cough Weight

- ☐ Most days
- ☐ Several days
- ☐ With chest infections

- ☐ Not at all

Question 2: I bring up phlegm (sputum)

- ☐ Most days
- ☐ Several days
- ☐ With chest infections
- ☐ Not at all

Question 3: I have shortness of breath

- ☐ Most days
- ☐ Several days
- ☐ Not at all

Question 4: I have attacks of wheezing

- ☐ Most days
- ☐ Several days
- ☐ A few days
- ☐ Only with chest infection
- ☐ Not at all

Question 5: How many attacks of chest trouble have you had

- ☐ 3 or more
- ☐ 1 or 2 attacks
- ☐ None

Question 6: How often do you have good days (with little chest trouble)?

- ☐ None
- ☐ A few
- ☐ Most are good
- ☐ Every day

Question 7: If you have a wheeze, is it worse in the morning?

- ☐ No
- ☐ Yes

Part 2

Question 8: How would you describe your chest condition?

- ☐ The most important problem I have
- ☐ Causes me a few problems
- ☐ Causes no problem

**Question 9:** Questions about what activities usually make you feel breathless	**Yes**	**No**
9a) Getting washed or dressed		
9b) Walking around the home		
9c) Walking outside on the level		
9d) Walking up a flight of stairs		
9e) Walking up hills		
**Question 10:** More questions about your cough and breathlessness	**Yes**	**No**
10a) My cough hurts		
10b) My cough makes me tired		
10c) I get breathless when I talk		
10d) I get breathless when I bend over		
10e) My cough or breathing disturbs my sleep		
10f) I get exhausted easily		

**Question 11:** Questions about other effects your chest trouble may have on you	**Yes**	**No**
11a) My cough or breathing is embarrassing in public<br>11b) My chest trouble is a nuisance to my family, friends or neighbors<br>11c) I get afraid or panic when I cannot get my breath<br>11d) I feel that I am not in control of my chest problem<br>11e) I have become frail or invalid because of my chest<br>11f) Exercise is not safe for me<br>11g) Everything seems too much of an effort		
**Question 12:** Questions about how activities may be affected by your breathing	**Yes**	**No**
12a) I take a long time to get washed or dressed<br>12b) I cannot take a bath or shower, or I take a long time<br>12c) I walk more slowly than other people, or I stop for rests<br>12d) If I walk up one flight of stairs, I have to go slowly or stop<br>13e) If I hurry or walk fast, I have to stop or slow down<br>12f) My breathing makes it difficult to do things such as walk up hills, carry things up stairs, light gardening such as weeding, dance, play bowls or play golf<br>12g) My breathing makes it difficult to do things such as carry heavy loads, dig the garden or shovel snow, jog or walk at 5 miles per hour, play tennis or swim		
**Question 13:** We would like to know how your chest trouble usually affects your daily life	**Yes**	**No**
13a) I cannot play sports or games<br>13b) I cannot go out for entertainment or recreation<br>13c) I cannot go out of the house to do the shopping<br>13d) I cannot do housework<br>13e) I cannot move far from my bed or chair		

Question 14: Tick the statement which you think best describes how your chest affects you

☐ It does not stop me doing anything I would like to do

☐ It stops me doing one or two things I would like to do

☐ It stops me doing most of the things I would like to do

☐ It stops me doing everything I would like to do
