# Supplementary material for: Management of COPD and Comorbidities in COPD patients by Dispensing Pharmaceutical Care following Global Initiative for chronic Obstructive Lung Disease-Guidelines (GOLD guidelines 2020): A study protocol for a Prospective Randomized Clinical Trial
Source: Heliyon. 2023 Oct 25;9(11):e21539. doi: 10.1016/j.heliyon.2023.e21539 (PMC10628705; doi:10.1016/j.heliyon.2023.e21539)
Supplement: Multimedia component 2 [file mmc2.pdf]

قائداعظم یونیورسٹی

QUAID-I-AZAM UNIVERSITY

Faculty of Biological Sciences
Bioethics Committee

No. #BEC-FBS-QAU2021-269 Dated: 29-03-2021

Dr. Amjad Khan,
Assistant Professor,
Department of Pharmacy,
Faculty of Biological Sciences,
Quaid-i-Azam University, Islamabad
45320, Pakistan

Subject:- **"Evaluation and Management of COPD and other Comorbidities in COPD Patients by Dispensing Pharmaceutical Care following the GOLD guidelines 2020: A Prospective Clinical Trial"**

Dear Dr. Amjad Khan,

We wish to inform you that your subject research study has been reviewed and is hereby granted approval for implementation by Bio-Ethical Committee (BEC) of Quaid-i-Azam University, Your study has been assigned protocol **#BEC-FBS-QAU2020-269.**

While the study is in progress, please inform us of any adverse events or new, relevant information about risks associated with the research. In case changes have to be made to the study procedure, the informed consent from and or informed consent process, the BEC must review and approve any of these changes prior to implementation.

Sincerely,

Prof. Dr. Sarwat Jahan
Department of Zoology

cc:
Dean, F.B.S
